# Sporadic Cryptosporidiosis Decline after Membrane Filtration of Public Water Supplies, England, 1996–2002

**DOI:** 10.3201/1102.040274

**Published:** 2005-02

**Authors:** Stella Goh, Mark Reacher, David P. Casemore, Neville Q. Verlander, André Charlett, Rachel M. Chalmers, Margaret Knowles, Anthony Pennington, Joy Williams, Keith Osborn, Sarah Richards

**Affiliations:** * Carlisle and District Primary Care Trust, Carlisle, United Kingdom;; † Health Protection Agency, Cambridge, United Kingdom;; ‡ University of Wales, Aberystwyth, Ceredigion, Wales, United Kingdom;; § Health Protection Agency Centre for Infections, Colindale, London, United Kingdom;; ¶ Singleton Hospital, Swansea, Wales, United Kingdom;; # Cumberland Infirmary, Carlisle, United Kingdom;; ** United Utilities, Great Sankey, Warrington, United Kingdom;; †† West Cumberland Hospital, Whitehaven, United Kingdom

**Keywords:** Cryptosporidium, Sporadic, Cryptosporidiosis, Diarrhea, Childhood, Incidence, Poisson Regression, Water treatment, Membrane filtration

## Abstract

The incidence of sporadic cryptosporidiosis among 106,000 residents of 2 local government districts in northwest England before and after installation of membrane filtration of public water supplies was compared to that of 59,700 residents whose public water supplies remained unchanged. A national outbreak of foot and mouth disease in livestock during 2001 was associated with a decline in sporadic human cryptosporidiosis in all regions of the United Kingdom. In a Poisson regression model, membrane filtration was associated with an estimated 79% reduction (incidence ratio 0.207, 95% confidence intervals 0.099–0.431, p < 0.0001) after adjustment for the interval of the foot and mouth disease epidemic and the water source. Despite the confounding effect of that epidemic, membrane filtration of the public water supply was effective in reducing the risk for sporadic human *Cryptosporidium* infection in this population.

## Abstract 2

Sporadic cryptosporidiosis and associated hospital admissions of children declined after membrane filtration of public drinking water supplies was introduced.

*Cryptosporidium* is a genus of enteric parasites that cause diarrhea in humans and many animal species worldwide; it is the third most common cause of nonviral infectious diarrhea reported in England and Wales (*1**,**2*). Oocysts are shed in large numbers in feces of infected humans and animals and contain highly infectious sporozoites when ingested (*1**,**3*). Disease may be prolonged and fatal in immunocompromised persons (*1*). *Cryptosporidium hominis* (previously designated *C. parvum* genotype 1) is found in humans but occurs naturally in livestock animals very rarely; *C. parvum* (previously designated *C. parvum* genotype 2) infects humans and livestock (*4**–**6*).

*Cryptosporidium* oocysts are a threat to the safety of drinking water supplies because they remain viable in water and damp soils for prolonged periods and are resistant to concentrations of disinfectants, including chlorine, used in conventional water treatment. Removal of these oocysts depends on sedimentation, coagulation, and filtration (*1**,**7**,**8*). We have previously reported a prospective case-control study of risk factors for sporadic cryptosporidiosis in residents of Allerdale and Copeland local government districts in North Cumbria, rural northwest England, from March 1, 1996, to February 29, 2000. That study showed a strong association with the usual daily volume of cold unboiled tap water drunk and with short visits to farms (*9*). We present extended observation of the population to August 31, 2002, during which time membrane filtration of public drinking water supplies was introduced for two thirds of the study population and a national outbreak of foot and mouth disease (FMD) in livestock occurred.

## Materials and Methods

The study area comprises part of the Lake District National Park. The lakes act as natural reservoirs for local public water supplies and have livestock farms and open grazing land abutting them. Approximately one third of the study population receive public water supplies from Ennerdale Lake, one third from Crummock Lake, and one third from a number of smaller sources. From March 1, 1996, to February 29, 2000, water from Ennerdale and Crummock Lakes was disinfected with chlorine, but unfiltered, because the low level of particulate matter in these sources precluded chemically assisted flocculation. Membrane filtration began on March l, 2000, at works treating water from Ennerdale and Crummock Lakes and remained active until the end of the study. The remaining third of the population received water from a number of smaller sources undergoing a variety of conventional treatments, including coagulation, filtration and chlorination, and chlorination alone. No changes in the treatment of water from these other sources occurred at any time.

### FMD Epidemic in Livestock

The first FMD case in livestock was confirmed on February 21, 2001, in southeast England and the last case on September 30, 2001, in northwest England (*10*). Epidemic controls were enforced throughout the United Kingdom; they included culling livestock, excluding livestock from traditional pastures, limiting livestock movements, and excluding the public from the countryside. The FMD epidemic was associated with marked attenuation of the usual spring peak in human cryptosporidiosis reporting from all regions of England and Wales and with a decline predominantly of *C. parvum* (livestock and human species). FMD epidemic controls were applied uniformly across the Allerdale and Copeland Districts and ended on January 21, 2002.

Analysis of risk factors and laboratory testing for *Cryptosporidium* were undertaken as previously described; cases with date of onset from March 1, 2000, to August 31, 2002, and associated controls were added (*5**,**9*). To determine if introduction of membrane filtration and the FMD epidemic in livestock were associated with a change in risk factors or incidence of human cryptosporidiosis, observations were divided into 5 intervals: before commissioning of membrane filtration (March 1, 1996, to February 29, 2000); membrane filtration commissioning (March 1, 2000, to July 31, 2000); established membrane filtration before the FMD epidemic (August 1, 2000, to February 20, 2001); the FMD livestock epidemic to ending of local FMD epidemic controls (February 21, 2001 to January 20, 2002); and post-FMD epidemic (January 21, 2002, to August 31, 2002).

### Case Definition

Case-patients were residents of Allerdale or Copeland who had diarrhea (>3 loose stools in a 24-hour period) with onset from March 1, 1996, to August 31, 2002; were fecal smear positive for *Cryptosporidium* oocysts, but feces negative for other enteric pathogens; and had spent at least 1 night within the study area in the 14 days before onset. Patients were excluded if, within 14 days of onset, they had contact with another household patient with cryptosporidiosis or any diarrhea illness, traveled outside the United Kingdom, or traveled within the United Kingdom and stayed outside the study area during the entire 14-day period before onset of illness; or if they, or a household member, had already been enrolled as a case-patient or control at any time during the study.

### Control Definition

Controls were residents of Allerdale or Copeland who had no history of diarrhea (defined as >3 loose stools in a 24-hour period) and had spent at least 1 night within the study area in the 14 days before interview. Potential controls were excluded if they had traveled outside the United Kingdom in the 14 days before the date of interview or if they had traveled within the United Kingdom and stayed outside the study area during the entire 14 day period before the date of interview. Potential controls were also excluded if they or a household member had already been enrolled as a case-patient or control at any time during the study.

The local water company provided details of the water sources, water supply zones, number of houses and residents served by each water source and water supply zone, and treatment of water to each zone. The study team and water company maintained vigilance for changes in the water supplies to the population at all stages of the study. In a minority of participants, the water source changed; these case-patients and controls were categorized as receiving mixed supplies from >1 source.

### Risk Factor Analysis

Five sets of contingency tables, 1 for each interval of observation defined by introduction of membrane filtration and the FMD epidemic, were constructed for each exposure variable, and odds ratios were calculated. Main effects variables were defined as those significant at p < 0.2 in any of the 5 sets of contingency tables. These variables—interval, age, sex, and water supply zone—were put into a multivariable model. Stepwise sequential removal of variables with p > 0.05 was undertaken retaining time interval, age, sex, and water supply zone. The significance of interaction terms between time interval and the remaining main effects variables was tested in separate models with stepwise sequential removal of terms with p > 0.05.

### Incidence Rates and Incident Rate Modeling

Incidence rates were determined for residents by water supply zone and modeled by Poisson regression using the number of cases as the predictor variable and the number of person-years of observation as the offset (*11**,**12*). The models had 3 predictor variables: membrane filtration (before and after), FMD epidemic (before, during, and after local FMD controls), and water source (Ennerdale, Crummock, and "other" water supplies). The interaction between these predictors was explored. The models provided estimates of the incidence rate ratio (IRR). The goodness-of-fit of the models was assessed. The species of *Cryptosporidium* isolates before and after membrane filtration were compared.

## Results

### Population and Water Supplies

Public water supplies derived from Crummock Lake served 58,295 residents; from Ennerdale Lake, 47,780 residents; and from a variety of other smaller water sources, including a few private water supplies, the remaining 59,699 residents of Allerdale and Copeland (Table A1). Public drinking water supplies derived from Crummock and Ennerdale Lakes before March 1, 2000, were chlorinated but not filtered (Table A1). Separate membrane filtration plants at water treatment works at Crummock and Ennerdale Lakes were commissioned from March 1, 2000, to July 31, 2000; full operation was achieved by August 1, 2000. These plants remained fully operational until the end of the study, August 31, 2002. The treatment of water derived from other sources remained unchanged for the study period. These multiple smaller sources received a variety of conventional treatments, including coagulation, filtration and chlorination, and chlorination alone. In addition, a small number of houses had private water supplies, which were untreated (Table A1).

### Recruitment and Exclusion of Patients

A total of 249 patients identified as having sporadic cryptosporidiosis were ascertained during the study period; 74 (30%) were excluded, and 175 (70%) were enrolled (Table 1). Of the 175 primary cases of cryptosporidiosis enrolled, 153 (87%) had onset dates from March 1, 1996, to February 29, 2000, before the commissioning of membrane filtration at Crummock and Ennerdale Lakes; 22 (13%) patients had onset from March 1, 2000, to August 31, 2002, after the membrane filtration plants were introduced (Table 1).

**Table 1:** Exclusions and recruitment of case-patients and controls

Exclusion criteria	n (%)		
	Before membrane filtration, March 1, 1996–February 29, 2000	After membrane filtration, March 1, 2000–August 31, 2002	Before and after membrane filtration, March 1, 1996–August 31, 2002
Case-patients			
Refused to participate	1 (0.5)	0 (0)	1 (0.4)
Could not complete adequate interview	1 (0.5)	1 (2.4)	2 (0.8)
Did not respond to letters or phone calls	2 (1.0)	0 (0)	2 (0.8)
Did not meet study case definition			
No history of diarrhea	1 (0.5)	1 (2.4)	2 (0.8)
Mixed enteric infection	1 (0.5)	0	1 (0.4)
Secondary case	36 (17.4)	10 (23.8)	46 (18.5)
Travel outside UK in 14 days before onset	8 (3.9)	8 (19.0)	16 (6.4)
Visitor to study area	1 (0.5)	0 (0)	1 (0.4)
Residence outside study area	1 (0.5)	0 (0)	1 (0.4)
Case-patient or household member previously interviewed as case or control	2 (1.0)	0 (0)	2 (0.8)
Potential case-patients approached	207 (100)	42 (100)	249 (100)
Potential cases excluded	54 (26.1)	20 (47.6)	74 (29.7)
Total case-patients enrolled	153 (73.9)	22 (52.4)	175 (70.3)
Controls			
Refused or unavailable for interview			
Refused to participate	23 (3.0)	12 (7.9)	35 (3.8)
Unavailable at requested interview times	125 (17.1)	23 (15.2)	148 (15.9)
Said interview times were not convenient	35 (4.5)	3 (2.0)	38 (4.1)
Address not found	3 (0.4)	0 (0)	3 (0.3)
Did not meet study control definition			
History of diarrhea	46 (5.9)	6 (4.0)	52 (5.6)
Travel outside UK in 14 days before interview	8 (1.0)	4 (2.6)	12 (1.3)
Not resident in study area in 14 days before interview	3 (0.4)	1 (0.7)	4 (0.4)
Moved from study area	27 (3.5)	7 (4.6)	34 (3.7)
Residence outside study area	2 (0.3)	0 (0)	2 (0.2)
Control or household member already interviewed as a case or control	7 (0.9)	1 (0.7)	8 (0.9)
Not enrolled for administrative reasons or reason not recorded			
Interview cancelled; 3 controls already enrolled for associated case	19 (2.4)	25 (16.6)	44 (4.7)
Interview cancelled; potential control found to be in wrong age group	9 (1.2)	0 (0)	9 (1.0)
Reason for exclusion not recorded	3 (0.4)	0 (0)	3 (0.3)
Potential controls approached	778 (100)	151 (100)	929 (100)
Potential controls excluded	310 (39.8)	82 (54.3)	392 (42.2)
Total controls enrolled	468 (60.2)	69 (45.7)	537 (57.8)

### Recruitment and Exclusion of Controls

A total of 929 potential controls were approached during the study; 392 (42%) were excluded, and 537 (58%) were enrolled. Two hundred and twenty one (24%) persons either refused to participate or were repeatedly unavailable for interview (Table 1). The address was not found for 3 (<1%). One hundred twelve (12%) were excluded because they did not meet the study control definition for a variety of reasons (Table 1). Fifty-six (6%) were not enrolled for administrative reasons. The study team cancelled interviews for 44 (5%) because 3 control interviews had been completed for the associated case and 9 (1%) interviews because the potential controls were found to be in the wrong age band; the reason for exclusion was not recorded for 3 potential controls (<1%). Of the 537 controls enrolled, 468 (87%) had interview dates from March 1, 1996, to February 29, 2000; and 69 (12.9%) had interviews from March 1, 2000, to August 31, 2002 (Table 1).

### Study Population

#### Patients

Of the 175 case-patients, 150 (86%) were <16 years of age, and 96 (55%) were <6 years of age. Ninety (51%) were male (Table 2). The proportion of cases <16 years of age and the proportion who were male were lower after membrane filtration was introduced into Crummock and Ennerdale Lake water. The proportion of case-patients served by water from other sources that never received membrane filtration was higher after introduction of membrane filtration (Table 2).

**Table 2:** Baseline characteristics of case-patients and controls

Characteristics	n (%)		
	Before membrane filtration, March 1, 1996–February 29, 2000	After membrane filtration, March 1, 2000–August 31, 2002	Before and after membrane filtration, March 1, 1996–August 31, 2002
Case-patients, total	153 (100)	22 (100)	175 (100)
Sex			
Female	70 (45.8)	15 (68.2)	85 (48.6)
Male	83 (54.2)	7 (31.8)	90 (51.4)
Age			
<1–5	87 (56.9)	9 (40.9)	96 (54.9)
6–15	47 (30.7)	7 (31.8)	54 (30.9)
16+	19 (12.4)	6 (27.3)	25 (14.3)
Water sources and water supply zones			
Crummock Lake			
Crummock North	37 (24.2)	2 (9.1)	39 (22.3)
Crummock South	19 (12.4)	4 (18.2)	23 (13.1)
Ennerdale Lake			
Ennerdale North	30 (19.6)	2 (9.1)	32 (18.3)
Ennerdale South	13 (8.5)	1 (4.5)	14 (8.0)
Other sources			
Millom	19 (12.4)	3 (13.6)	22 (12.6)
Quarry Hill	16 (10.5)	6 (27.3)	22 (12.6)
Hausegill	3 (2.0)	0	3 (1.7)
Hayknott	2 (1.3)	0	2 (1.1)
Underscar	1 (0.7)	2 (9.1)	3 (1.7)
Fellside	0	0	0
Mixed >1 source	11 (7.2)	1 (4.5)	12 (6.9)
Different private water supplies	2 (1.3)	1 (4.5)	3 (1.7)
Controls, total	468 (100)	69 (100)	537 (100)
Sex			
Female	234 (50)	31 (44.9)	265 (49.3)
Male	234 (50)	38 (55.1)	272 (50.7)
Age			
<1–5	273 (58.3)	27 (39.1)	300 (55.9)
6–15	137 (29.3)	20 (29.0)	157 (29.2)
16+	58 (12.4)	12 (17.4)	70 (13.0)
Water sources and water supply zones			
Crummock Lake			
Crummock North	104 (22.2)	7 (10.1)	111 (20.7)
Crummock South	49 (10.5)	13 (18.8)	62 (11.5)
Ennerdale Lake			
Ennerdale North	100 (21.4)	7 (10.1)	107 (19.9)
Ennerdale South	43 (9.2)	3 (4.3)	46 (8.6)
Other sources			
Millom	54 (11.5)	10 (14.5)	64 (11.9)
Quarry Hill	42 (9.0)	16 (23.2)	58 (10.8)
Hausegill	5 (1.1)	0	5 (0.9)
Hayknott	6 (1.3)	1 (1.4)	7 (1.3)
Underscar	3 (0.6)	6 (8.7)	8 (1.5)
Bridgend	0	2 (2.9)	2 (0.4)
Fellside	1 (0.2)	0	1 (0.2)
Mixed*	51 (10.9)	4 (5.8)	55 (10.2)
Different private water supplies	10 (2.1)	0	10 (1.9)

*Mixed: mixed supply derived from Ennerdale and Crummock, or from Ennerdale and another source, or from Crummock and another source.

In addition to diarrhea, a substantial proportion of patients had abdominal pain, vomiting, fever, loss of appetite, and weight loss (Table A2). Forty-two (24%) patients remained symptomatic at interview. Of the 133 (76%) whose symptoms had abated at interview, the median duration of illness was 9 days (range 2–21 days) (Table A2). In children <6 years of age, 14 (25%) of 55 of boys and 6 (15%) of 41 girls were admitted to hospital because of diarrhea (Table A3). The admission rates in children 6–15 years of age were 3 (11%) of 28 boys and 3 (12%) of 26 girls. Most of the patients and all male patients <6 years of age had onset dates before the membrane filtration was introduced (Table A3). Twenty-six (17%) of the 150 patients who were <16 years of age were admitted to hospital, but none over this age.

Species identification was undertaken for 68 fecal specimens from patients with onset from January 1, 1998, to February 29, 2000 (Table A4). Fifty-seven (84%) were *C. parvum*. Thirteen (81%) of the 16 smears derived from patients with onset dates from March 1, 2000, to August 31, 2002, were also *C. parvum*. Overall, 70 (83.3%) of the 84 specimens for which the species was identified were *C. parvum*.

#### Controls

The 537 controls had similar age, sex, and drinking water supplies as the 175 patients (Table 2). The time between notification of a case by a microbiology laboratory to the study-coordinating center to enrollment of the patient and his or her associated controls was a median of 2 weeks (range 1–8 weeks) and was similar before and after the introduction of membrane filtration.

### Risk Factor Analysis

None of the interaction terms between the main effects variables and time intervals of observation, defined by introduction of membrane filtration and the FMD epidemic in livestock, were significant, including the term for the usual volume of cold unboiled tap water drunk per day (p = 0.12). These interaction terms were therefore excluded from the final multivariable risk factor model (Table 3). The risk for sporadic cryptosporidiosis was independently associated with the usual volume of cold unboiled tap water drunk each day, with contact with cattle farms and noncattle farms, and with feeding pets leftovers. Water supply zones, the time interval of observation, age, and gender were not independently associated with having a case (Table 3).

**Table 3:** Final multivariable model of risk factors for sporadic cryptosporidiosis, Allerdale and Copeland residents, March 1, 1996, to August 31, 2002

Risk factors	Case-patients	Controls	Adjusted odds ratio*	Lower 95% CI†	Upper 95% CI	p value
Sex						
Female	85	265	1	0.45	1.184	0.202
Male	90	272	0.73			
Age						
<1–5	96	300	1.002/y	0.938	1.021	0.872
6–15	54	157				
16+	25	70				
Water sources and water supply zones						0.556
Crummock Lake						
Crummock North	39	111	1			
Crummock South	23	62	1.262	0.547	2.913	
Ennerdale Lake						
Ennerdale North	32	107	1.25	0.594	2.63	
Ennerdale South	14	46	0.556	0.164	1.881	
Other sources						
Millom	22	64	1.502	0.686	3.288	
Quarry Hill	22	58	0.703	0.273	1.81	
Hausegill	3	5	1.356	0.22	8.34	
Ayknott	2	7	1.016	0.1	10.29	
Underscar	3	9	0.983	0.1	9.18	
Fellside	0	1	0.005	0	∞	
Bridgend	0	2	0.004	0	∞	
Mixed public supplies	12	55	0.893	0.32	2.494	
Private water supplies	3	10	0.1	0.007	1	
Usual daily volume of cold unboiled tap water drunk at home			1.543 per pint	1.212	1.965	< 0.001
<1/4 pint	25	122				
1/4–1 pint	78	260				
>1–2 pints	46	106				
>2 pints	22	42				
Contact with a cattle farm						
Yes	19	29	4.532	1.757	11.69	0.002
No	144	475				
Contact with a noncattle farm						
Yes	17	24	3.809	1.677	8.651	0.002
No	146	471				
Feed pet leftovers						
Yes	14	19	3.746	1.214	11.56	0.021
No	161	515				
Interval of study						0.585
March 1, 1996–February 29, 2000	153	468	1			
March 1, 2000–July 31, 2000	4	16	0.965	0.235	3.958	
August 1, 2000–February 20, 2001	6	16	1.115	0.319	3.895	
February 21, 2001–January 21, 2002	6	18	0.367	0.078	1.72	
January 11, 2002–August 31, 2002	6	19	0.485	0.138	1.701	

*Adjusted for accidentally touching animal feces, feeding pets biscuits, feeding pets raw vegetables, contact with anyone outside the household with a history of diarrhea, type of sewage system to the house, consumption of mixed salad, and local authority of residence. †CI, confidence interval.

### Incidence and Seasonality

**Figure 1:**
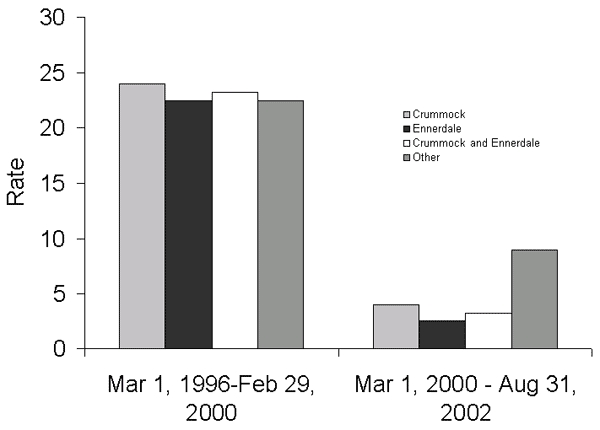
Cases of primary cryptosporidiosis per 100,000 person-years before and after membrane filtration introduced into public water supplies, derived from Crummock Lake, Ennerdale Lake, and other water sources.

**Figure 2:**
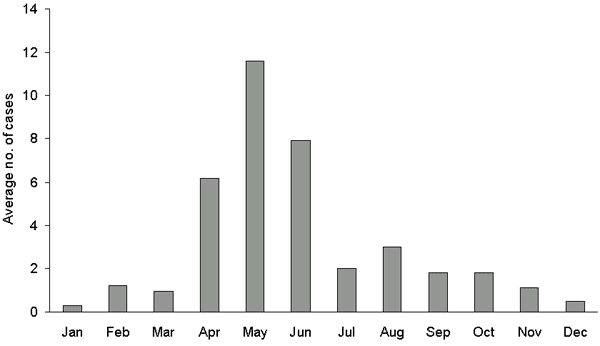
Average number of cases by month of onset before membrane filtration, March 1, 1996–February 29, 2000, Allerdale and Copeland local government districts.

**Figure 3:**
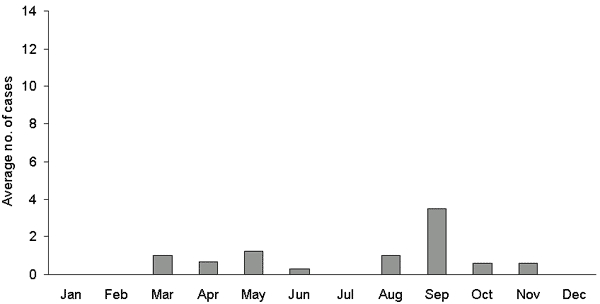
Average number of cases by month of onset after membrane filtration introduced March 1, 2000, to August 31, 2002, Allerdale and Copeland local government districts.

The incidence within the populations served by public water supplies derived from Ennerdale Lake, Crummock Lake, and other water sources was similar before March 2000 at ≈22 cases per 100,000 person years but declined to <10 per 100,000 person years after March 1, 2000 (Table 4) (Figure 1). The decline was more marked in the populations served by water derived from treatment works at Ennerdale and Crummock Lakes, where membrane filtration plants had been installed, than in the population served by other water sources, where membrane filtration was not installed. A well-defined spring peak in cases was apparent from 1996 to 1999, but not from 2000 to 2002 (Figures 2 and 3).

**Table 4:** Incidence of sporadic cryptosporidiosis by water source, March 1, 1996–August 31, 2002

Water source and time intervals*	Membrane filtration (MF)	Cases (n)	Person-years	Rate per 100,000 person-years	95% CI†
Crummock Lake					
Before MF interval	No	56	233,623	23.97	(18.11, 31.13)
After MF interval					
Commissioning MF	Yes	1	25,726	3.89	(0.10, 21.66)
Established MF pre-FMDE	Yes	1	33,970	2.94	(0.07, 16.40)
Established MF and FMDE	Yes	1	54,902	1.82	(0.05, 10.15)
Established MF and post-FMDE	Yes	3	31,490	9.53	(1.96, 27.84)
Total after MF	Yes	6	146,088	4.11	(1.51, 8.91)
Ennerdale Lake					
Before MF interval	No	43	191,053	22.51	(16.29, 30.32)
After MF interval					
Commissioning MF	Yes	0	20,258	0	(0, 18.21)
Established MF pre-FMDE	Yes	1	27,223	3.67	(0.09, 20.47)
Established MF and FMDE	Yes	2	46,387	4.31	(0.52, 15.57)
Established MF and post-FMDE	Yes	0	26,606	0	(0, 13.86)
Total after MF	yes	3	120,474	2.49	(0.51, 7.28)
Other sources					
Before MF interval	No	54	238,265	22.66	(17.03, 29.57)
After MF interval					
Commissioning MF	No	3	24,449	12.27	(2.53, 35.86)
Established MF pre-FMDE	No	4	32,257	12.4	(3.38 , 31.75 )
Established MF and FMDE	No	3	51,997	5.77	(1.19, 16.86)
Established MF and post-FMDE	No	3	29,824	10.06	(2.07, 29.40)
Total after MF	No	13	138,527	9.38	(5.00,16.05)

*Time intervals. pre-MF (membrane filtration) March 1, 1996–February 29, 2000; post-MF March 1, 2000–August 31, 2002. Post-MF comprises the following: commissioning MF, March 1, 2000–July 31, 2000; established MF before foot and mouth disease epidemic (FMDE), August 1, 2000–February 20, 2001; established MF and FMDE, February 21, 2001–January 20, 2002; established MF and post-FMDE, January 21, 2002–August31, 2002. †CI, confidence intervals.

After membrane filtration was introduced for Crummock and Ennerdale supplies, an estimated reduction in incidence of ≈79% occurred (IRR 0.207, 95% confidence intervals [CI] 0.099–0.431), p < 0.0001, after adjustment for the FMD epidemic interval and water source in a Poisson regression model (Table 5). The decrease attributed to the FMD interval was ≈60% (IRR 0.394, 95% CI 0.167–0.925), with some evidence of a residual effect after the end of local FMD epidemic controls (IRR 0.686, 95% CI 0.292–1.61). No additional effect was contributed by water source (p = 0.6). The data for this model are presented graphically in Figure 1 and detailed in Table 4. Little difference was made by modeling the intervals for commissioning and postcommissioning of membrane filtration separately, and no significant difference was seen between these rate estimates when this modeling was done (p = 0.35)

**Table 5:** Poisson regression model of the incidence of sporadic cryptosporidiosis*

Predictor	Category	IRR	95% CI	p
Membrane filtration	No	Reference		
	Yes	0.207	0.099–0.431	< 0.0001
Foot and mouth disease epidemic	Pre	Reference		
	During	0.394	0.167–0.925	
	Post	0.686	0.292–1.612	0.05
Water supply	Crummock	Reference		
	Ennerdale	0.907	0.620–1.329	
	Other	0.820	0.573–1.174	0.6

*IRR, incidence rate ratio; CI, confidence interval. Goodness-of-fit test (chi square 10.84, 9 df, p = 0.3).

## Discussion

Consumption of cold unboiled tap water from public drinking water supplies was shown to be a leading independent risk factor for sporadic cryptosporidiosis with a highly significant increase in risk with the usual volume drunk each day (Table 3). Risk was also increased by contact with cattle farms and noncattle farms and with feeding pets leftovers. Fifty five percent of patients were <6 years of age, and 31% were 6–15 years. Infection was predominantly with *C. parvum* (livestock and human species). The results of the risk factor analysis for the entire study period were similar to those obtained for the interval before installation of membrane filtration, when most cases arose (*9*). Illness was prolonged and almost one fifth of children <6 years of age required hospital admission. The excess in hospitalization in boys <6 years of age may suggest that young boys are more vulnerable to *Cryptosporidium* than young girls, a bias in favor of admitting young boys, or a combination of these factors.

The 2001 FMD epidemic in livestock, which occurred after membrane filtration was introduced in Allerdale and Crummock Lake water, affected all regions of the United Kingdom (*10*). This livestock epidemic was associated with a highly significant decline in laboratory reports of human cryptosporidiosis from all regions of England and Wales and was more marked in Northwest England. The decline in reports was most marked for *C. parvum* (the species infectious in humans and livestock species) than for C. hominis (infectious only in humans) (*10*). The FMD epidemic control measures of excluding the public from the countryside, extensively culling farm animals, and limiting animal movements probably decreased direct and indirect exposure of the human population to livestock and livestock feces.

The annual agricultural and horticultural census conducted by the U.K. Department for Environment, Food and Rural Affairs and its predecessor, the Ministry of Agriculture, Food and Fisheries showed >600,000 sheep, 300,000 lambs, 100,000 total cattle and calves, and 40,000 calves <1 year of age in each of the years 1996–2000 within the 135,000 hectares of agricultural land in Allerdale and Copeland local government districts (*13**,**14*). A substantial decline occurred in 2001 and 2002 associated with the FMD epidemic, but no evidence suggested that the decline in animal densities or change in human contact with livestock and with the countryside differed within Allerdale and Copeland, according to the sources or distribution of the public drinking water supplies (*13**,**14*). The decline in incidence attributable to the FMD epidemic effect was therefore expected for our entire study population, regardless of its household water supply. We therefore believe that the experience of the population served by other supplies provided a valid measure of the impact of the FMD epidemic in livestock, whereas the population served by water from Ennerdale and Crummock Lakes experienced the effect of both membrane filtration and the FMD epidemic. The results of the Poisson regression model indicated a marked reduction of incidence in sporadic cryptosporidiosis following introduction of membrane filtration after adjustment for the FMD epidemic interval and water source (Table 5). Despite the confounding effect of the FMD epidemic, our study provides convincing evidence that membrane filtration was highly effective in reducing the risk for sporadic cryptosporidiosis in this population; this measure was also associated with a decline in hospital admissions for cryptosporidiosis in children, especially of boys <6 years of age.

The incidence rates associated with other supplies from a number of different sources and treatment works, some using conventional flocculation and filtration, were similar from March 1996 to February 2001 to the rates in the population served by Crummock and Ennerdale Lakes, whose water was unfiltered at this time. This finding supports the notion that conventional sand filtration and flocculation may be insufficient to prevent intermittent low-level *Cryptosporidium* oocyst contamination of treated water. The local water company has since closed higher risk sources and substituted them with water from lower risk catchments.

Our observations strongly support recent revision of the UK drinking water regulations requiring water companies to undertake risk assessments of water sources, and where judged to be a risk, to implement continuous monitoring of *Cryptosporidium* oocyst concentrations in treated water (*15*). A minimum standard of an average of <1 oocyst per 10 L of water in any 24-hour period is required.

The substantial negative impact of waterborne cryptosporidiosis leading to potentially life-threatening diarrhea and stunting in childhood is well-recognized in developing countries (*16**,**17*). Our findings show that *Cryptosporidium* remains an obstacle in water and sanitation infrastructure and a threat to child health in industrialized countries as well. The scale of this effect will continue to be underestimated if adequate surveillance of cryptosporidiosis by testing diarrheal feces specimens for *Cryptosporidium* and collation of positive test results, especially in children, is omitted by health services (*18*). Although the study population was located in an area of livestock farming with high historic rates of cryptosporidiosis in England, the demonstration that public drinking water supplies were a leading independent risk factor for sporadic cryptosporidiosis and that introduction of membrane filtration at water treatment works was effective in substantially lowering this risk, may have relevance to water companies, regulators, policymakers, and consumers in other countries.

## Appendix

**Table A1:** Water sources, water distribution zones, filtration, and populations served, Copeland and Allerdale Local Authorities, 1996–2002

Water sources	Water zones	Filtration of water supplies	Average population
Crummock Lake	Crummock North Crummock South	Unfiltered March 1, 1996–February 29, 2000; membrane filtration from March 1, 2000, to August 31, 2002	58,295
Ennerdale Lake	Ennerdale North Ennerdale South	Unfiltered March 1, 1996–February 29, 2000; membrane filtration from March 1, 2000, to August 31, 2002	47,780
"Other*"	Millom Quarry Hill Hausegill Hayknott Underscar Bridgend Fellside Multiple sources Private	Conventionally filtered* throughout the study period (March 1, 1996–August 31, 2002)	59,699

*A variety of conventionally treated and unfiltered water, and private supplies.

**Table A2:** Symptoms and duration of symptoms in case-patients

Characteristics	n (%)		
	Before membrane filtration	After membrane filtration	Before and after membrane filtration
	March 1, 1996–February 29, 2000	March 1, 2000–August 31, 2002	March 1, 1996–August 31, 2002
Symptoms			
Diarrhea	153 (100)	22 (100)	175 (100)
Vomiting	94 (61.4)	10 (45.5)	104 (59.4)
Abdominal pain	110 (71.9)	18 (81.8)	128 (73.1)
Fever	69 (45.1)	9 (40.9)	78 (44.6)
Loss of appetite	68 (44.4)	18 (81.8)	86 (49.1)
Weight loss	56 (36.6)	17 (77.3)	73 (41.7)
Symptoms at interview	38 (24.8)	4 (18.2)	42 (24.0)
No symptoms at interview	115 (75.2)	18 (81.8)	133 (76.0)
Median days* symptomatic	9	10	9
Range	2–21	3–20	2–21

*Of patients without symptoms at interview.

**Table A3:** Admissions to hospital by age and sex

Characteristics	Before membrane filtration			After membrane filtration			Before and after membrane filtration		
	March 1, 1996–February 29, 2000			March 1, 2000–August 31, 2002			March 1, 1996–August 31, 2002		
	Total	Admitted	%	Total	Admitted	%	Total	Admitted	%
Female									
<1–5 y	34	5	15	7	1	14	41	6	15
6–15 y	22	2	9	4	1	25	26	3	12
Total >1–15 y	56	7	13	11	2	18	67	9	13
Male									
<1–5 y	53	14	26	2	0	0	55	14	25
6–15 y	25	2	8	3	1	33	28	3	11
Total <1–15 y	78	16	21	5	1	20	83	17	20
F and M									
<1–5 y	87	19	22	9	1	11	96	20	21
6–15 y	47	4	9	7	2	29	54	6	11
Total <1–15 y	134	23	17	16	3	19	150	26	17

**Table A4:** Speciation of *Cryptosporidium* isolates

Isolates	n (%)		
	Before membrane filtration	After membrane filtration	Before and after membrane filtration
	January 1, 1998–February 29, 2000	March 1, 2000–August 31, 2002	January 1, 1998–August 31, 2002
Total speciated	68 (100)	16 (100)	84 (100)
*C. hominis*	1 (1.5)	3 (18.8)	4 (4.8)
*C. parvum*	57 (83.8)	13 (81.2)	70 (83.3)
Not determined	10 (14.7)	0 (0)	10 (11.9)
